# Addendum guidelines for the prevention of peanut allergy in the United States: report of the National Institute of Allergy and Infectious Diseases-sponsored expert panel

**DOI:** 10.1186/s13223-016-0175-4

**Published:** 2017-01-06

**Authors:** Alkis Togias, Susan F. Cooper, Maria L. Acebal, Amal Assa’ad, James R. Baker, Lisa A. Beck, Julie Block, Carol Byrd-Bredbenner, Edmond S. Chan, Lawrence F. Eichenfield, David M. Fleischer, George J. Fuchs, Glenn T. Furuta, Matthew J. Greenhawt, Ruchi S. Gupta, Michele Habich, Stacie M. Jones, Kari Keaton, Antonella Muraro, Marshall Plaut, Lanny J. Rosenwasser, Daniel Rotrosen, Hugh A. Sampson, Lynda C. Schneider, Scott H. Sicherer, Robert Sidbury, Jonathan Spergel, David R. Stukus, Carina Venter, Joshua A. Boyce

**Affiliations:** 1 The National Institute of Allergy and Infectious Diseases, Bethesda, MD USA; 2 The Board of Directors, Food Allergy Research & Education, McLean, VA USA; 3 The Division of Allergy and Immunology, Cincinnati Children’s Hospital Medical Center, University of Cincinnati, Cincinnati, OH USA; 4 Food Allergy Research & Education, McLean and the Division of Allergy and Clinical Immunology, University of Michigan Health System, Ann Arbor, MI USA; 5 The Department of Dermatology, University of Rochester Medical Center, New York, USA; 6 The National Eczema Association, San Rafael, CA USA; 7 The Department of Nutritional Sciences, Rutgers University, NB, Canada; 8 The Division of Allergy and Immunology, Department of Pediatrics, BC Children’s Hospital, University of British Columbia, Vancouver, BC Canada; 9 The Departments of Dermatology and Pediatrics, University of California, San Diego School of Medicine, Rady Children’s Hospital, San Diego, CA USA; 10 The Section of Allergy and Immunology, Department of Pediatrics, Children’s Hospital Colorado, University of Colorado Denver School of Medicine, Aurora, CO USA; 11 The Division of Gastroenterology, Hepatology and Nutrition, Department of Pediatrics, University of Kentucky College of Medicine, Kentucky Children’s Hospital, Lexington, KY USA; 12 The Digestive Health Institute, Children’s Hospital Colorado, Aurora, CO USA; 13 The Division of Academic General Pediatrics and Primary Care, Department of Pediatrics, The Ann and Robert H. Lurie Children’s Hospital of Chicago, Northwestern University Feinberg School of Medicine, Chicago, IL USA; 14 Northwestern Medicine, Central DuPage Hospital, Winfield, IL USA; 15 The Division of Allergy and Immunology, Department of Pediatrics, University of Arkansas for Medical Sciences, Arkansas Children’s Hospital, Little Rock, AR USA; 16 Metro DC Food Allergy Support Group, Rockville, MD USA; 17 The Food Allergy Referral Centre, Department of Women and Child Health, Padua University Hospital, Padua, Italy; 18 University of Missouri-Kansas City School of Medicine, Kansas City, MO USA; 19 The Division of Allergy and Immunology, Department of Pediatrics, Harvard Medical School, Boston, MA USA; 20 The Division of Pediatric Allergy and Immunology, Icahn School of Medicine at Mount Sinai, New York, USA; 21 The Division of Allergy and Immunology, Boston Children’s Hospital, Boston, MA USA; 22 The Department of Pediatrics, Division of Dermatology, Seattle Children’s Hospital, University of Washington School of Medicine, Seattle, WA USA; 23 The Division of Allergy and Immunology, Department of Pediatrics, Children’s Hospital of Philadelphia, Perelman School of Medicine at University of Pennsylvania, Philadelphia, PA USA; 24 The Department of Pediatrics, Section of Allergy and Immunology, Nationwide Children’s Hospital, Ohio State University College of Medicine, Columbus, OH USA; 25 The Division of Allergy and Immunology, Cincinnati Children’s Hospital Medical Center, Cincinnati, OH USA; 26 The Departments of Medicine and Pediatrics, Harvard Medical School, Boston, MA USA

**Keywords:** Food, Peanut, Allergy, Prevention, Guidelines

## Abstract

**Background:**

Food allergy is an important public health problem because it affects children and adults, can be severe and even life-threatening, and may be increasing in prevalence. Beginning in 2008, the National Institute of Allergy and Infectious Diseases, working with other organizations and advocacy groups, led the development of the first clinical guidelines for the diagnosis and management of food allergy. A recent landmark clinical trial and other emerging data suggest that peanut allergy can be prevented through introduction of peanut-containing foods beginning in infancy.

**Objectives:**

Prompted by these findings, along with 25 professional organizations, federal agencies, and patient advocacy groups, the National Institute of Allergy and Infectious Diseases facilitated development of addendum guidelines to specifically address the prevention of peanut allergy.

**Results:**

The addendum provides 3 separate guidelines for infants at various risk levels for the development of peanut allergy and is intended for use by a wide variety of health care providers. Topics addressed include the definition of risk categories, appropriate use of testing (specific IgE measurement, skin prick tests, and oral food challenges), and the timing and approaches for introduction of peanut-containing foods in the health care provider’s office or at home. The addendum guidelines provide the background, rationale, and strength of evidence for each recommendation.

**Conclusions:**

Guidelines have been developed for early introduction of peanut-containing foods into the diets of infants at various risk levels for peanut allergy.

## Background

Peanut allergy is a growing public health problem. In 1999, peanut allergy was estimated to affect 0.4% of children and 0.7% of adults in the United States [1], and by 2010, peanut allergy prevalence had increased to approximately 2% among children in a national survey [2], with similar results reported in a regional cohort [3]. Peanut allergy is the leading cause of death related to food-induced anaphylaxis in the United States [4, 5], and although overall mortality is low, the fear of life-threatening anaphylactic reactions contributes significantly to the medical and psychosocial burden of disease. In the majority of patients, peanut allergy begins early in life and persists as a lifelong problem. Therefore, cost-effective measures to prevent peanut allergy would have a high effect in terms of improving public health, reducing personal suffering, and decreasing health care use and costs.

The “Guidelines for the diagnosis and management of food allergy in the United States” [6] were published in December 2010 by an expert panel and a Coordinating Committee convened by the National Institute of Allergy and Infectious Diseases (NIAID). These guidelines did not offer strategies for the prevention of food allergy and particularly peanut allergy because of a lack of definitive studies at the time. The guidelines indicated that “insufficient evidence exists for delaying introduction of solid foods, including potentially allergenic foods, beyond 4–6 months of age, even in infants at risk of developing allergic disease.” This statement differed from previous clinical practice guidelines in the United Kingdom [7] and United States, [8] which recommended the exclusion of allergenic foods from the diets of infants at high risk for allergy and is consistent with more recent recommendations regarding primary allergy prevention [9–12].

In February 2015, the New England Journal of Medicine published the results of the Learning Early about Peanut Allergy (LEAP) trial [13]. This trial was based on a prior observation [14] that the prevalence of peanut allergy was tenfold higher among Jewish children in the United Kingdom compared with Israeli children of similar ancestry. In Israel, peanut-containing foods are usually introduced in the diet when infants are approximately 7 months of age and consumed in substantial amounts, whereas in the United Kingdom children do not typically consume any peanut-containing foods during their first year of life. The LEAP trial randomized 640 children between 4 and 11 months of age with severe eczema, egg allergy, or both to consume or avoid peanut-containing foods until 60 months of age, at which time a peanut oral food challenge (OFC) was conducted to determine the prevalence of peanut allergy. LEAP trial participants were stratified at study entry into 2 separate study cohorts on the basis of pre-existing sensitization to peanut, as determined by means of skin prick testing: one cohort consisted of infants with no measureable skin test wheal to peanut (negative skin test response) and the other consisted of those with measurable wheal responses (1–4 mm in diameter). Infants with a 5 mm wheal diameter or greater were not randomized because the majority of infants at this level of sensitization were presumed to be allergic to peanut. Among the 530 participants in the intention-to-treat population with negative baseline skin test response to peanut, the prevalence of peanut allergy at 60 months of age was 13.7% in the peanut avoidance group and 1.9% in the peanut consumption group (P < .001; an 86.1% relative reduction in the prevalence of peanut allergy). Among the 98 participants with a measurable peanut skin test response at entry, the prevalence of peanut allergy was 35.3% in the avoidance group and 10.6% in the consumption group (P = .004; a 70% relative reduction in the prevalence of peanut allergy).

The LEAP trial was the first randomized trial to study early allergen introduction as a preventive strategy. Because of the size of the observed effect and the large number of study participants, its outcome received wide publicity in both the medical community and the press. This raised the need to operationalize the LEAP findings by developing clinical recommendations focusing on peanut allergy prevention. To achieve this goal and its wide implementation, the NIAID invited the members of the 2010 Guidelines Coordinating Committee and other stakeholder organizations to develop this addendum on peanut allergy prevention to the 2010 “Guidelines for the diagnosis and management of food allergy in the United States.” Twenty-six stakeholder organizations participated in this 2015–2016 Coordinating Committee. Of note, unrelated to this effort, a consensus statement on behalf of 9 international professional societies regarding the implications and implementation of the LEAP trial findings was published as well [15].

Additional evidence on early introduction of allergenic foods comes from the LEAP-On study [16], which demonstrated the durability of oral tolerance to peanut achieved in the LEAP trial and the enquiring about tolerance study [17], which assessed the potential benefits of early introduction of 6 allergenic foods in a non–high-risk cohort.

## Development of the 2017 addendum to the 2010 “Guidelines for the diagnosis and management of food allergy”

The process to develop the 2017 addendum closely followed that used in the 2010 guidelines [6].

### Coordinating committee

The NIAID established a Coordinating Committee (CC), the members of which are listed in Appendix A, to oversee the development of the addendum; review drafts of the addendum for accuracy, practicality, clarity, and broad utility of the recommendations in clinical practice; review and approve the final addendum; and disseminate the addendum. The CC members represented 26 professional organizations, advocacy groups, and federal agencies.

### Expert panel

The CC convened an expert panel (EP) in June 2015 that was chaired by Joshua Boyce, MD. The 26 panel members, listed in Appendix B, were specialists from a variety of relevant clinical, scientific, and public health areas. Panel members were nominated by the CC organizations, and the composition of the panel received unanimous approval by the CC member organizations.

The charge to the EP was to use the literature review prepared by the NIAID (see the next section) in conjunction with consensus expert opinion and EP-identified supplementary documents to (1) develop evidence-based recommendations for the early introduction of dietary peanut to prevent peanut allergy; (2) agree on principles for grading the evidence; (3) achieve consensus while allowing ample opportunity for consideration of divergent opinions; (4) determine whether the recommendations could extend beyond peanut to other food allergens; and (5) keep patient and societal interests at the forefront. The new recommendations are intended to supplement and modify guidelines 37 to 40 in Section 5.3.4 of the 2010 guidelines: “Prevention of food allergy.”

### Literature review

NIAID staff conducted a literature search of PubMed limited to the years 2010 (January) to 2016 (June). Using the following specific search terms ([food allergy or milk allergy or egg allergy or peanut allergy] OR [eczema or atopic dermatitis] AND prevention), PubMed returned more than 1500 articles. NIAID staff reviewed 1506 abstracts and assessed each for relevance to the topic of food allergy prevention with an emphasis on peanut allergy. Sixty-four publications (original research articles, editorials/letters, and systematic reviews) were deemed relevant and placed into 2 tiers: tier 1 contained 18 items considered highly relevant to the early introduction of peanut or other allergenic foods (see Appendix C), and tier 2 contained 46 items on related topics, such as food allergy or eczema prevention.

### Assessing the quality of the body of evidence

For each of the 18 tier 1 references, the EP assessed quality by using the Grading of Recommendations Assessment, Development and Evaluation (GRADE) approach [18]. GRADE provides a comprehensive and transparent methodology to develop recommendations for the diagnosis, treatment, and management of patients. In assessing the body of evidence of a group of relevant articles or of a single article, GRADE considers study design and other factors, such as the precision, consistency, and directness of the data. By using this approach, GRADE then provides a categorical assessment of the contribution of individual publications and the overall quality and strength of the body of evidence.

Each publication was assigned a grade according to the following criteria [19, 20]:

- High: further research is very unlikely to have an effect on the quality of the body of evidence, and therefore the confidence in the recommendation is high and unlikely to change.
- Moderate: further research is likely to have an effect on the quality of the body of evidence and may change the recommendation.
- Low: further research is very likely to have an important effect on the body of evidence and is likely to change the recommendation.

A GRADE designation of “low” for the quality of evidence does not imply that an article is not factually correct or lacks scientific merit. For example, a well-designed and executed single-site study of a treatment in a small cohort of highly selected subjects may still yield an overall GRADE rating of “low.” This is because such a study is characterized as providing “sparse” data, and the patient population may not be representative of the at-risk population. Each of these factors reduces the level of evidence from “high,” which is the initial designation for evidence from randomized controlled trials. It is worth emphasizing that these 2 limitations are not of the study per se but of the body of evidence.

### Preparation of the draft addendum

The draft version of the addendum, prepared by the NIAID, contained 3 new guidelines and was reviewed, modified, and endorsed by the EP members. The EP-approved document was forwarded to the CC members for review.

### Public comment period, addendum revision, and final approval

Concurrent with CC member review, the draft addendum was posted to the NIAID Web site in March 2016 for a period of 45 days to allow for public review and comment. One hundred four comments were received. All comments were reviewed by the EP and the CC, and some contributed to the final revision of the addendum. The final addendum was reviewed and approved by the EP and the CC.

### Dissemination of the addendum guidelines

The final addendum is published herein and available through the Internet.

## Defining the strength of each clinical guideline

The EP has used the verb “recommends” or “suggests” for each clinical recommendation.

These words convey the strength of the recommendation, defined as follows:

- *Recommend* is used when the EP strongly recommended for or against a particular course of action.
- *Suggest* is used when the EP weakly recommended for or against a particular course of action.

## Addendum guidelines

Table 1 provides a summary of the 3 addendum guidelines to be used as a quick reference.

**Table 1:** Summary of addendum guidelines 1, 2, and 3

Addendum guideline	Infant criteria	Recommendations	Earliest age of peanut introduction
1	Severe eczema, egg allergy, or both	Strongly consider evaluation by sIgE measurement and/or SPT and, if necessary, an OFC. Based on test results, introduce peanut-containing foods	4–6 months
2	Mild-to-moderate eczema	Introduce peanut-containing foods	Around 6 months
3	No eczema or any food allergy	Introduce peanut-containing foods	Age appropriate and in accordance with family preferences and cultural practices

The EP came to consensus on the following 3 definitions used throughout the addendum guidelines.

- *Severe eczema* is defined as persistent or frequently recurring eczema with typical morphology and distribution assessed as severe by a health care provider and requiring frequent need for prescription-strength topical corticosteroids, calcineurin inhibitors, or other anti-inflammatory agents despite appropriate use of emollients.
- *Egg allergy* is defined as a history of an allergic reaction to egg and a skin prick test (SPT) wheal diameter of 3 mm or greater with egg white extract, or a positive oral egg food challenge result.
- A *specialist* is defined as a health care provider with the training and experience to (1) perform and interpret SPTs and OFCs and (2) know and manage their risks. Such persons must have appropriate medications and equipment on site.

### Addendum guideline 1

The EP recommends that infants with severe eczema, egg allergy, or both have introduction of age-appropriate peanut-containing food as early as 4–6 months of age to reduce the risk of peanut allergy. Other solid foods should be introduced before peanut-containing foods to show that the infant is developmentally ready. The EP recommends that evaluation with peanut-specific IgE (peanut sIgE) measurement, SPTs, or both be strongly considered before introduction of peanut to determine if peanut should be introduced and, if so, the preferred method of introduction. To minimize a delay in peanut introduction for children who may test negative, testing for peanut sIgE may be the preferred initial approach in certain health care settings, such as family medicine, paediatrics, or dermatology practices, in which skin prick testing is not routine. Alternatively, referral for assessment by a specialist may be an option if desired by the health care provider and when available in a timely manner.

Figure 1 provides recommended approaches for evaluation of children with severe eczema, egg allergy, or both before peanut introduction.

**Fig. 1:**
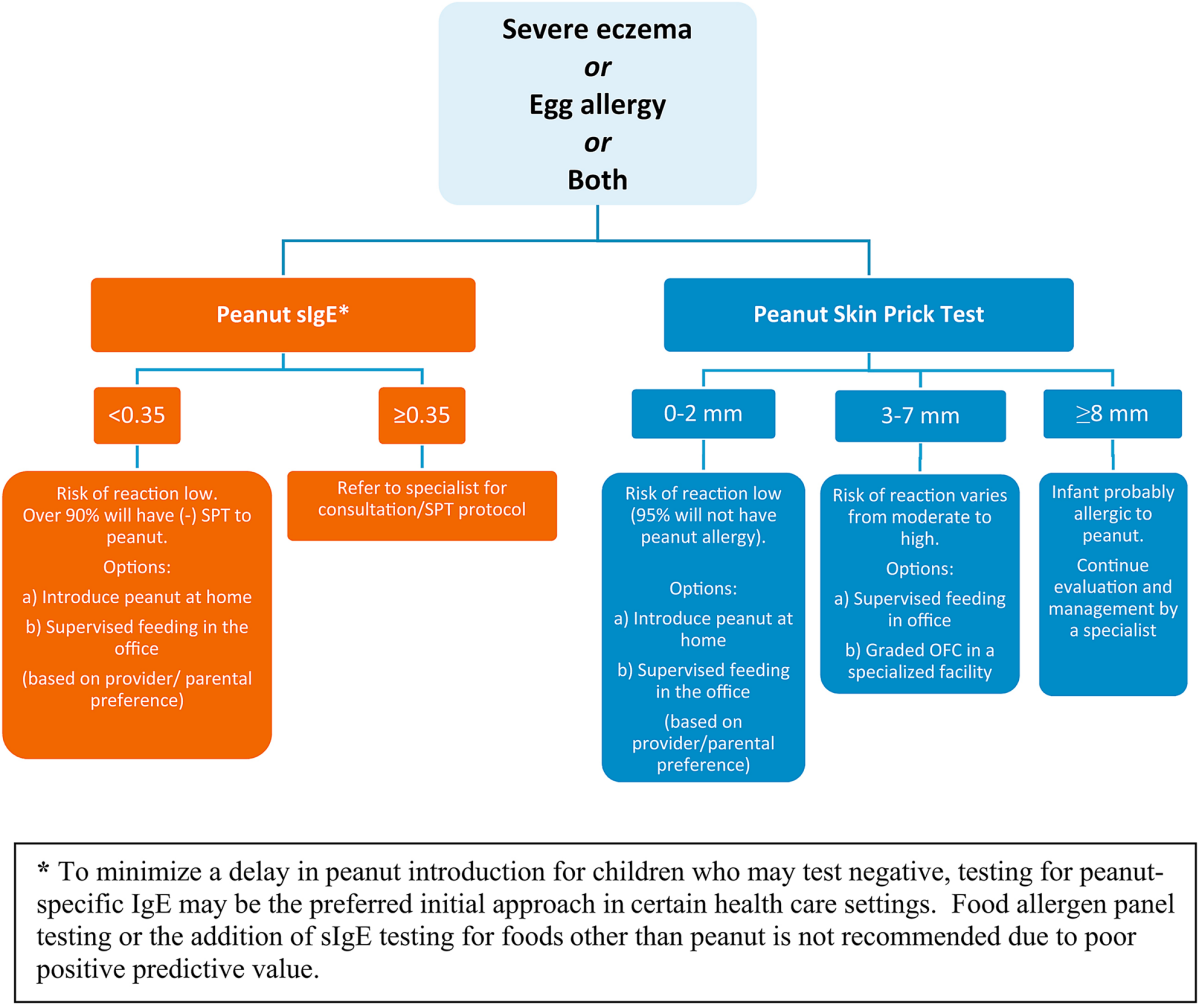
Recommended approaches for evaluation of children with severe eczema and/or egg allergy before peanut introduction

A peanut sIgE level of less than 0.35 kUA/L has strong negative predictive value for the diagnosis of peanut allergy [21]. Therefore, peanut sIgE testing may help in certain health care settings (eg, family medicine, paediatric, or dermatology practices, where skin prick testing is not routine) to reduce unnecessary referrals of children with severe eczema, egg allergy, or both and to minimize a delay in peanut introduction for children who may have negative test results. However, the EP emphasizes that a peanut sIgE level of 0.35 kUA/L or greater lacks adequate positive predictive value for the diagnosis of peanut allergy, and an infant with a value of 0.35 kUA/L or greater should be referred to a specialist.

Thus, peanut sIgE testing can place an infant into one of 2 categories (Fig. 1):

- sIgE Category A: If the peanut sIgE level is less than 0.35 kUA/L (ImmunoCAP), the EP recommends that peanut should be introduced in the diet soon thereafter, with a cumulative first dose of approximately 2 g of peanut protein given in this feeding. This can be given as a feeding at home (Appendix D), considering the low likelihood of a severe allergic reaction. If the caregiver or health care provider has concerns, a supervised feeding can be offered at the health care provider’s office (Appendix E).
- sIgE Category B: If the peanut sIgE level is 0.35 kUA/L or greater (ImmunoCAP), the EP recommends that the child be referred to a specialist for further consultation and possible skin prick testing.

The EP does not recommend food allergen panel testing or the addition of sIgE testing for foods other than peanut because of their poor positive predictive value, which could lead to misinterpretation, overdiagnosis of food allergy, and unnecessary dietary restrictions [6].

SPTs with peanut extract can place an infant in one of 3 categories (Fig. 1):

- SPT Category A: If an SPT to peanut extract produces a wheal diameter of 2 mm or less above saline control, the EP recommends that peanut be introduced in the diet soon after testing, with a cumulative first dose of approximately 2 g of peanut protein given in this feeding. This can be given at home (Appendix D), considering the low likelihood of a severe allergic reaction. If the caregiver or health care provider has concerns, a supervised feeding can be offered at the health care provider’s office (Appendix E).
- SPT Category B: If an SPT to peanut extract produces a wheal diameter of 3 to 7 mm greater than that elicited by the saline control, the EP suggests that a supervised peanut feeding or a graded OFC be undertaken at a specialist’s office or a specialized facility (see Appendices E and G, respectively). Infants in this category can be sensitized without being allergic to peanut and might benefit from early peanut consumption. If the supervised peanut feeding or graded OFC yields no reaction, the EP recommends that peanut should be added to the child’s diet. If the supervised peanut feeding or the graded OFC results in an allergic reaction, the EP recommends that the child should strictly avoid dietary peanut and the family should be counselled regarding food allergy management.
- SPT Category C: If an SPT produces a wheal diameter 8 mm or greater than that elicited by the saline control, the likelihood of peanut allergy is high. Children in this category should continue to be evaluated and managed by a specialist [21–23].

**Box 1:** Important considerations for skin prick testing

SPT reagents, testing devices, and methodology can differ significantly among health care providers in the United States or elsewhere.22 The EP recommends that specialists adjust their SPT categorization criteria according to their own training and experience. Health care providers conducting OFCs in infants with 3 mm or greater SPT responses should be aware that the probability of a positive challenge response increases with wheal size. These data come from the HealthNuts Study in children 12 to 18 months of age; of note, the severity of these reactions was relatively mild [21, 23].			

#### How much dietary peanut protein to introduce

If the decision is made to introduce dietary peanut based on the recommendations of addendum guideline 1, the total amount of peanut protein to be regularly consumed per week should be approximately 6 to 7 g over 3 or more feedings (see Appendix F). In the LEAP trial, at evaluations conducted at 12 and 30 months of age, 75% of children in the peanut consumption group reported eating at least this amount of peanut, based on analysis of a 3-day food diary recorded just before the evaluation.

#### Rationale

Infants with severe eczema, egg allergy, or both are at high risk for the development of peanut allergy. Significant evidence on this group is available from the infants who participated in the LEAP trial or were screened for the LEAP trial but were not enrolled because of a large SPT response (>4 mm). At 60 months of age, approximately 23% of peanut avoiders and those infants not enrolled had food allergy [24].

#### Balance of benefits and harms

In the LEAP trial, among the 530 participants in the intention-to-treat population with negative baseline SPT responses to peanut, 13.7% of the avoidance group and 1.9% of the consumption group had peanut allergy at 60 months of age (P < .001; a 12.6% absolute risk reduction and an 86.1% relative risk reduction in the prevalence of peanut allergy, resulting in a number needed to treat of 8.5 [number of infants needed to have early introduction of peanut to prevent peanut allergy in one child]). Among the 98 participants with positive peanut SPT responses at entry, 35.3% of the avoidance group and 10.6% of the consumption group had peanut allergy at 60 months of age (P = .004; a 24.7% absolute risk reduction and a 70% relative risk reduction in the prevalence of peanut allergy, resulting in a number needed to treat of 4).

The LEAP-on study [24] demonstrated that the benefits achieved in the LEAP trial persisted when LEAP trial peanut consumers subsequently avoided peanut for 1 year from 60 to 72 months of age. This indicates that the oral tolerance achieved in the LEAP trial was durable.

The LEAP trial did not include infants with SPT wheals greater than 4 mm, and therefore no data are available on the potential effectiveness of peanut consumption in preventing peanut allergy in this group. However, EP members believe it is possible that some of these infants may benefit from early introduction of peanut provided that they tolerate oral peanut.

As shown in Fig. 1, the EP recommends that infants with severe eczema, egg allergy, or both, with peanut sIgE levels of less than 0.35 kUA/L or with a peanut SPT wheal of 2 mm or less have dietary peanut introduced as early as 4–6 months of age without a need for further evaluation. This recommendation is supported by expert opinion and analysis of the LEAP population findings. In the LEAP trial, infants consuming peanut in this post hoc defined category had a relative risk reduction of 79% of having peanut allergy at 60 months of age compared with infants who avoided peanut.

In the LEAP trial, at study entry, all infants randomly assigned to the consuming group had a baseline peanut OFC. Of the 272 infants with no wheal induced by peanut SPT and who received a baseline oral peanut challenge, only 1 had a reaction presenting as an erythematous urticarial rash that was graded as a “moderate” adverse event and was treated successfully with chlorpheniramine. Among the 29 infants with a wheal diameter of 1–2 mm who received a baseline oral peanut challenge, 2 had reactions, which also presented with mild symptoms not requiring treatment with epinephrine. Therefore, for the SPT Category A children, the risk of a severe reaction to peanut at first introduction is low, and introduction of peanut at home is an option. However, it is understandable that some caregivers of infants with severe eczema, egg allergy, or both may be uncomfortable introducing dietary peanut at home. In such cases the health care provider should offer the option of a supervised feeding of a peanut-containing food in the office.

The rate of positive peanut OFC results at baseline for infants with a 3–4 mm wheal diameter (4/17 infants) was higher than in infants with 0 to 2 mm wheal diameters (3/301 infants), but the elicited symptoms were mild. Infants with larger wheal diameters (>4 mm) were not included in the LEAP trial, and therefore no safety data are available from this group. However, based on the Australian HealthNuts study, which conducted peanut OFCs in a large number of older (12–18 months old) children from the general Australian population, the rate of reactions to peanut is expected to be substantially higher with increasing SPT wheal diameter [21, 23]. In the HealthNuts study [23] an SPT wheal diameter of 8 mm or greater had a 95% positive predictive value for peanut allergy (positive oral peanut challenge result). Therefore, the EP recommends that for SPT Category B infants (3–7 mm SPT wheal diameter), a supervised feeding or a graded peanut OFC should be conducted in a specialist’s office or a specialized facility (Appendix G). SPT Category C infants are considered high risk for established allergy to peanut and should not receive peanut-containing foods in their diet, unless such foods are recommended by a specialist after further evaluation.

#### Quality of evidence: moderate

The designation of the quality of evidence as “moderate” (as opposed to “high”) is based on the fact that this recommendation derives primarily from a single randomized, open-label study: the LEAP trial. However, it should be noted that the assessment of the LEAP trial’s primary outcome was based on a double-blind, placebo-controlled OFC. Furthermore, confidence in this recommendation is bolstered by the large effect size demonstrated in the LEAP trial and prior epidemiologic data that peanut allergy is relatively infrequent in Israel, where early childhood consumption of peanut is common.

#### Contribution of expert opinion

Significant.

#### Additional comments

1. Breast-feeding recommendations: the EP recognizes that early introduction of peanut may seem to depart from recommendations for exclusive breast-feeding through 6 months of age [25, 26]. However, it should be noted that data from the nutrition analysis of the LEAP cohort [27] indicate that introduction of peanut did not affect the duration or frequency of breast-feeding and did not influence growth or nutrition.
2. Age of peanut introduction: for children with severe eczema, egg allergy, or both, the EP recommends that introduction of solid foods begins at 4–6 months of age, starting with solid food other than peanut, so that the child can demonstrate the ability to consume solid food without evidence of nonspecific signs and symptoms that could be confused with IgE-mediated food allergy. However, it is important to note that infants in the LEAP trial were enrolled between 4 and 11 months of age and benefitted from peanut consumption regardless of age at entry. Therefore, if the 4- to 6-month time window is missed for any reason, including developmental delay, infants may still benefit from early peanut introduction. On the other hand, older age at screening is associated with larger wheal diameters induced by peanut SPT and hence a higher likelihood of established peanut allergy [28].
  A practical consideration for applying this guideline at 4–6 months of age is that infants visit their health care provider for well-child evaluations and infant immunizations at this time. This provides a fortuitous opportunity for eczema evaluation, caregiver reporting of egg allergy, and, if needed, referral to a specialist for peanut allergy evaluation before dietary introduction of peanut.
3. Considerations for family members with established peanut allergy: the EP recognizes that many infants eligible for early peanut introduction under this guideline will have older siblings or caregivers with established peanut allergy. The EP recommends that in this situation caregivers discuss with their health care providers the overall benefit (reduced risk of peanut allergy in the infant) versus risk (potential for further sensitization and accidental exposure of the family member to peanut) of adding peanut to the infant’s diet.
4. Children identified as allergic to peanut: for children who have been identified as allergic to peanut, the EP recommends strict peanut avoidance. This may include those children in SPT Category B who fail the supervised peanut feeding or the OFC, or those children in SPT Category C who, on further evaluation by a specialist, are confirmed as being allergic to peanut. These children should be under long-term management by a specialist.

### Addendum guideline 2

The EP suggests that infants with mild-to-moderate eczema should have introduction of age-appropriate peanut-containing food around 6 months of age, in accordance with family preferences and cultural practices, to reduce the risk of peanut allergy. Other solid foods should be introduced before peanut-containing foods to show that the infant is developmentally ready. The EP recommends that infants in this category may have dietary peanut introduced at home without an in-office evaluation. However, the EP recognizes that some caregivers and health care providers may desire an in-office supervised feeding, evaluation, or both.

#### Rationale

The LEAP trial did not target infants with mild or moderate eczema. The EP considered the potential risk/benefit ratio of early dietary peanut introduction in infants with mild-to-moderate eczema and concluded that the individual and societal benefits of introducing peanut in this population would be significant. The EP has no reason to believe that the mechanisms of protection of early dietary peanut differ in infants with mild-to-moderate eczema from those that lead to protection in infants at higher risk of peanut allergy.

#### Balance of benefits and harms

The LEAP trial included only infants with severe eczema or egg allergy based on careful medical history. Therefore, some infants who participated in the LEAP trial based on the presence of egg allergy had atopic dermatitis severity scores (SCORAD scores [29]) at screening that would have placed them in the moderate or mild eczema category. The EP considered the outcomes of these children and concluded that infants with mild-to-moderate eczema would likely benefit from early peanut introduction.

#### Quality of evidence

Low.

The quality of evidence is low because this recommendation is based on extrapolation of data from a single study.

#### Contribution of expert opinion

Significant.

#### Additional comment

Additional support for early introduction of peanut in infants who do not have severe eczema comes from the Enquiring About Tolerance study [17], which enrolled infants from the general population at 3 months of age and sequentially introduced 6 allergenic foods beginning at the time of enrolment. These children were not intentionally selected based on increased risk of food allergy or atopy. Although the intention-to-treat group did not show benefit, most likely because of relatively poor compliance with feeding recommendations, the children in the per-protocol group who had peanut introduced early in infancy showed a significant reduction in peanut sensitization and peanut allergy at age 3 years. This study also provides support for guideline 3 below.

### Addendum guideline 3

The EP suggests that infants without eczema or any food allergy have age-appropriate peanut-containing foods freely introduced in the diet together with other solid foods and in accordance with family preferences and cultural practices.

#### Rationale

No evidence exists for restricting allergenic foods in infants without known risks for food allergy. The probability for development of peanut allergy in such children is very low. However, approximately 14% of all children with peanut allergy at age 12–18 months in the HealthNuts Study lacked known risk factors for food allergy [16]. Consequently, because such children constitute a significant majority of any birth cohort, they contribute substantially to the overall societal burden of peanut allergy. The EP finds no evidence to suggest that mechanisms of oral tolerance induction would differ in these infants from the immunologic mechanisms that are protective in infants at higher risk of peanut allergy. Thus, the early introduction of dietary peanut in children without risk factors for peanut allergy is generally anticipated to be safe and to contribute modestly to an overall reduction in the prevalence of peanut allergy. Furthermore, in countries such as Israel, where peanut products are a popular component of the diet and where they are introduced early in life, the prevalence of peanut allergy is low [14].

#### Balance of benefits and harms

The EP acknowledges that any analysis of benefit and harm in this population relies primarily on expert opinion and is subject to current differences in regional/societal rates of peanut consumption and peanut sensitization. In countries where peanut products are not widely consumed by adults, early dietary introduction of peanut could lead to an increase in sensitization and allergic manifestations. Hence the EP cautions that this guideline be implemented in the context of societal routines/norms.

#### Quality of evidence

Low.

#### Contribution of expert opinion

Significant.

**Box 2:** Clinical implications

These guidelines will help health care providers with early introduction of peanut-containing foods in infants at various risk levels for peanut allergy. Early introduction of peanut will result in the prevention of peanut allergy in a large number of infants.			

## Appendix A: Coordinating Committee member organizations and representatives

**Academy of Nutrition and Dietetics**

http://www.eatright.org/

Alison Steiber, PhD, RD

**Allergy & Asthma Network Mothers of Asthmatics (AANMA)**

http://www.allergyasthmanetwork.org/main/

Tonya A. Winders, MBA

**American Academy of Allergy, Asthma & Immunology (AAAAI)**

https://www.aaaai.org/home.aspx

Hugh A. Sampson, MD

David Fleischer, MD

**American Academy of Family Physicians (AAFP)**

http://www.aafp.org/home.html

Jason Matuszak, MD

**American Academy of Dermatology (AAD)**

https://www.aad.org/

Lawrence F. Eichenfield, MD, FAAD

Jon Hanifin, MD

**American Academy of Emergency Medicine (AAEM)**

http://www.aaem.org/

Joseph P. Wood, MD, JD

**American Academy of Pediatrics (AAP)**

https://www.aap.org

Scott H. Sicherer, MD, FAAP

**American Academy of Physician Assistants (AAPA)**

https://www.aapa.org/

Gabriel Ortiz, MPAS, PA-C, DFAAPA

**American College of Allergy, Asthma and Immunology (ACAAI)**

http://acaai.org/

Amal Assa’ad, MD

**American College of Gastroenterology (ACG)**

http://gi.org/

Steven J. Czinn, MD, FACG

**American Partnership for Eosinophilic Disorders (APFED)**

http://apfed.org/

Wendy Book, MD

**American Society for Nutrition (ASN)**

http://www.nutrition.org/

George J. Fuchs III, MD

**Asthma and Allergy Foundation of America (AAFA)**

http://www.aafa.org/

Meryl Bloomrosen, MBA, MBI

David R. Stukus, MD

**Canadian Society of Allergy and Clinical Immunology (CSACI)**

http://www.csaci.ca/

Edmond Chan, MD, FRCPC

**Eunice Kennedy Shriver National Institute of Child Health & Human Development (NICHD)**

https://www.nichd.nih.gov

Gilman Grave, MD

**European Academy of Allergy and Clinical Immunology (EAACI)**

http://www.eaaci.org/

Antonella Muraro, MD, PhD

**Food Allergy Research & Education (FARE)**

https://www.foodallergy.org/

James R. Baker, MD

Mary Jane Marchisotto

**National Eczema Association (NEA)**

http://nationaleczema.org/

Julie Block

**National Heart, Lung, and Blood Institute (NHLBI)**

http://www.nhlbi.nih.gov/

Janet M. de Jesus, MS, RD

**National Institute of Allergy and Infectious Diseases (NIAID)**

http://www.niaid.nih.gov/

Daniel Rotrosen, MD

Alkis Togias, MD

Marshall Plaut, MD

**National Institute of Arthritis and Musculoskeletal and Skin Diseases (NIAMS)**

http://www.niams.nih.gov/

Ricardo Cibotti, PhD

**National Institute of Diabetes and Digestive and Kidney Diseases (NIDDK)**

www.niddk.nih.gov

Frank Hamilton, MD, MPH

Margaret A. McDowell, PhD, MPH, RD (retired)

Rachel Fisher, MS, MPH, RD

**North American Society for Pediatric Gastroenterology, Hepatology and Nutrition (NASPGHAN)**

http://www.naspghan.org/

Glenn Furuta, MD

**Society of Pediatric Nurses (SPN)**

http://www.pedsnurses.org/

Michele Habich, DNP, APN/CNS, CPN

**United States Department of Agriculture (USDA)**

http://www.usda.gov/

Soheila J. Maleki, PhD

**World Allergy Organization (WAO)**

http://www.worldallergy.org/

Lanny J. Rosenwasser, MD

## Appendix B. Expert Panel, June 2015

*Chair*

**Joshua A. Boyce, MD**

Professor of Medicine and Pediatrics

Harvard Medical School

Director, Inflammation and Allergic Disease Research Section

Director, Jeff and Penny Vinik Center for Allergic Disease Research

Specialty: Allergy/pediatric pulmonology

*Panelists*

**Maria Acebal, JD**

Board of Directors, Food Allergy Research & Education

Member of NIAID Advisory Council

Former CEO of Food Allergy and Anaphylaxis Network

Specialty: Advocacy

**Amal Assa’ad, MD**

Professor, University of Cincinnati Department of Pediatrics

Director, FARE Center of Excellence in Food Allergy

Director of Clinical Services, Division of Allergy and Immunology

Associate Director, Division of Allergy and Immunology

Cincinnati Children’s Hospital Medical Center

Specialty: Allergy/pediatrics

**James R. Baker, Jr, MD**

CEO and Chief Medical Officer

Food Allergy Research & Education, McLean VA

Founding Director, Mary H. Weiser Food Allergy Center, University of Michigan

Professor of Internal Medicine, Division of Allergy and Clinical Immunology

University of Michigan Health System

Specialty: Allergy/advocacy/education

**Lisa A. Beck, MD**

Professor, Department of Dermatology

University of Rochester Medical Center

School of Medicine and Dentistry

Specialty: Dermatology

**Julie Block**

President and CEO

National Eczema Association

Specialty: Advocacy/education

**Carol Byrd-Bredbenner, PhD, RD, FAND**

Professor of Nutrition/Extension Specialist

Rutgers University, School of Environmental and Biological Sciences

Specialty: Nutrition/health communication/behavioral science

**Edmond S. Chan, MD, FRCPC**

Clinical Associate Professor

Head, Division of Allergy and Immunology

Department of Pediatrics

BC Children’s Hospital

University of British Columbia

Specialty: Allergy/pediatrics

**Lawrence F. Eichenfield, MD**

Professor of Pediatrics and Dermatology

Chief, Pediatric and Adolescent Dermatology

Rady Children’s Hospital, San Diego

University of California, San Diego School of Medicine

Specialty: Dermatology/pediatrics

**David M. Fleischer, MD**

Associate Professor of Pediatrics

University of Colorado School of Medicine

Children’s Hospital Colorado, Aurora, CO

Specialty: Allergy/pediatrics

**George J. Fuchs III, MD**

Professor of Pediatrics

University of Kentucky College of Medicine

Chief, Gastroenterology, Nutrition & Hepatology

Kentucky Children’s Hospital

Specialty: Gastroenterology/pediatrics

**Glenn T. Furuta, MD**

Professor of Pediatrics

Director, Gastrointestinal Eosinophilic Diseases Program

University of Colorado School of Medicine

Children’s Hospital Colorado, Aurora, CO

Specialty: Gastroenterology/pediatrics

**Matthew J. Greenhawt, MD, MBA, MSc**

Assistant Professor of Pediatrics

Allergy Section

University of Colorado School of Medicine

Children’s Hospital Colorado, Aurora, CO

Specialty: Allergy/pediatrics

**Ruchi Gupta, MD, MPH**

Associate Professor of Pediatrics and Medicine

Director, Food Allergy Outcomes Research Program

Ann and Robert H. Lurie Children’s Hospital of Chicago

Northwestern Medicine, Northwestern University

Specialty: Pediatrics

**Michele Habich, DNP, APN/CNS, CPN**

Advanced Practice Nurse

Northwestern Medicine, Central DuPage Hospital

Specialty: Nursing/pediatrics/education

**Stacie M. Jones, MD**

Professor of Pediatrics

University of Arkansas for Medical Sciences

Chief, Allergy and Immunology

Arkansas Children’s Hospital

Specialty: Allergy/pediatrics

**Kari Keaton**

Facilitator, Metro DC Food Allergy Support Group

Specialty: Advocacy/education

**Antonella Muraro, MD, PhD**

President of European Academy of Allergy and Clinical Immunology (EAACI)

Professor of Allergy and Pediatric Allergy

Head of the Veneto Region Food Allergy Centre of Excellence for Research and Treatment

University Hospital of Padua, Italy

Specialty: Allergy/pediatrics

**Lanny J. Rosenwasser, MD**

Immediate Past President, World Allergy Organization

Professor of Medicine

University of Missouri-Kansas City-School of Medicine

Specialty: Allergy/pediatrics

**Hugh A. Sampson, MD**

Professor of Pediatrics, Allergy and Immunology

Icahn School of Medicine at Mount Sinai

Director, Jaffe Food Allergy Institute

Specialty: Allergy/pediatrics

**Lynda C. Schneider, MD**

Professor of Pediatrics

Harvard Medical School

Director, Allergy Program

Boston Children’s Hospital

Specialty: Allergy/pediatrics

**Scott H. Sicherer, MD**

Professor Pediatrics, Allergy and Immunology

Icahn School of Medicine at Mount Sinai

Division Chief, Pediatric Allergy and Immunology

Specialty: Allergy/pediatrics

**Robert Sidbury, MD, MPH**

Professor

Department of Pediatrics

Chief, Division of Dermatology

Seattle Children’s Hospital

University of Washington School of Medicine

Specialty: Dermatology/pediatrics

**Jonathan Spergel, MD, PhD**

Stuart Starr Professor of Pediatrics

Chief, Allergy Section

Director, Center for Pediatric Eosinophilic Disorders

The Children’s Hospital of Philadelphia

Perelman School of Medicine, University of Pennsylvania

Specialty: Allergy/pediatrics

**David R. Stukus, MD**

Assistant Professor of Pediatrics

Section of Allergy/Immunology

Nationwide Children’s Hospital

Columbus

Specialty: Allergy/pediatrics

**Carina Venter, PhD, RD**

Allergy Specialist, Dietitian

Cincinnati Children’s Hospital Medical Center

University of Cincinnati College of Medicine

Specialty: Allergy/dietitian/pediatrics

## Appendix C: Tier 1 references

- Feeney M, Du Toit G, Roberts R, Sayre PH, Lawson K, Bahnson HT, et al. Impact of peanut consumption in the LEAP study: feasibility, growth and nutrition. J Allergy Clin Immunol 2016;138:1108-18.
- Koplin JJ, Peters RL, Dharmage SC, Gurrin L, Tang MLK, Ponsonby AL, et al. Understanding the feasibility and implications of implementing early peanut introduction for prevention of peanut allergy. J Allergy Clin Immunol 2016;138:1131-41.e2.
- Perkin MR, Logan K, Tseng A, Raji B, Ayis S, Peacock J, et al. Randomized trial of introduction of allergenic foods in breast-fed infants. N Engl J Med 2016;374:1733-43.
- Du Toit G, Sayre PH, Roberts G, Sever ML, Lawson K, Bahnson HT, et al. Effect of avoidance on peanut allergy after early peanut consumption. N Engl J Med 2016;374:1435-43.
- Chang YS, Trivedi MK, Jha A, Lin YF, Dimaano L, García-Romero MT. Synbiotics for prevention and treatment of atopic dermatitis: a meta-analysis of randomized clinical trials. JAMA Pediatr 2016;170:236-42.
- O’Connor C, Kelleher M, O’B Hourihane J. Calculating the effect of populationlevel implementation of the Learning Early About Peanut Allergy (LEAP) protocol to prevent peanut allergy. J Allergy Clin Immunol 2016;137:1263-4.e2.
- Grimshaw KE, Bryant T, Oliver EM, Martin J, Maskell J, Kemp T, et al. Incidence and risk factors for food hypersensitivity in UK infants: results from a birth cohort study. Clin Transl Allergy 2016;6:1.
- Rabinovitch N, Shah D, Lanser BJ. Look before you LEAP: risk of anaphylaxis in high-risk infants with early introduction of peanut. J Allergy Clin Immunol 2015;136:822.
- Peters RL, Allen KJ, Dharmage SC, Lodge CJ, Koplin JJ, Ponsonby AL, et al. Differential factors associated with challenge-proven food allergy phenotypes in a population cohort of infants: a latent class analysis. Clin Exp Allergy 2015;45:953-63.
- Peters RL, Allen KJ, Dharmage SC, Koplin JJ, Dang T, Tilbrook KP, et al. Natural history of peanut allergy and predictors of resolution in the first 4 years of life: a population-based assessment. J Allergy Clin Immunol 2015;135:1257-66.e2.
- Du Toit G, Roberts G, Sayre PH, Bahnson HT, Radulovic S, Santos AF, et al. Randomized trial of peanut consumption in infants at risk for peanut allergy. N Engl J Med 2015;372:803-13.
- Martin PE, Eckert JK, Koplin JJ, Lowe AJ, Gurrin LC, Dharmage SC, et al. Which infants with eczema are at risk of food allergy? Results from a population-based cohort. Clin Exp Allergy 2015;45:255-64.
- Grimshaw KE, Maskell J, Oliver EM, Morris RC, Foote KD, Mills EN, et al. Introduction of complementary foods and the relationship to food allergy. Pediatrics 2013;132:e1529-38.
- Palmer DJ, Metcalfe J, Makrides M, Gold MS, Quinn P, West CE, et al. Early regular egg exposure in infants with eczema: a randomized controlled trial. J Allergy Clin Immunol 2013;132:387-92.e1.
- Du Toit G, Roberts G, Sayre PH, Plaut M, Bahnson HT, Mitchell H, et al. Identifying infants at high risk of peanut allergy: the Learning Early About Peanut Allergy (LEAP) screening study. J Allergy Clin Immunol 2013;131:135-43, e1-12.
- Joseph CL, Ownby DR, Havstad SL, Woodcroft KJ, Wegienka G, MacKechnie H, et al. Early complementary feeding and risk of food sensitization in a birth cohort. J Allergy Clin Immunol 2011;127:1203-10.e5.
- Koplin JJ, Osborne NJ, Wake M, Martin PE, Gurrin LC, Robinson MN, et al. Can early introduction of egg prevent egg allergy in infants? A population-based study. J Allergy Clin Immunol 2010;126:807-13.
- Katz Y, Rajuan N, Goldberg MR, Eisenberg E, Heyman E, Cohen A, et al. Early exposure to cow’s milk protein is protective against IgE-mediated cow’s milk protein allergy. J. Allergy Clin Immunol 2010;126:77-82.e1.

## Appendix D. Instructions for home feeding of peanut protein for infants at low risk of an allergic reaction to peanut

These instructions for home feeding of peanut protein are provided by your doctor. You should discuss any questions that you have with your doctor before starting. These instructions are meant for feeding infants who have severe eczema or egg allergy and were allergy tested (blood test, skin test, or both) with results that your doctor considers safe for you to introduce peanut protein at home (low risk of allergy).

### General instructions

1. Feed your infant only when he or she is healthy; do not do the feeding if he or she has a cold, vomiting, diarrhea, or other illness.
2. Give the first peanut feeding at home and not at a day care facility or restaurant.
3. Make sure at least 1 adult will be able to focus all of his or her attention on the infant, without distractions from other children or household activities.
4. Make sure that you will be able to spend at least 2 h with your infant after the feeding to watch for any signs of an allergic reaction.

### Feeding your infant

1. Prepare a full portion of one of the peanut-containing foods from the recipe options below.
2. Offer your infant a small part of the peanut serving on the tip of a spoon.
3. Wait 10 min.
4. If there is no allergic reaction after this small taste, then slowly give the remainder of the peanut-containing food at the infant’s usual eating speed.

#### What are symptoms of an allergic reaction? What should I look for?

- Mild symptoms can include:
  - a new rash
    or
  - a few hives around the mouth or face
- More severe symptoms can include any of the following alone or in combination:
  - lip swelling
  - vomiting
  - widespread hives (welts) over the body
  - face or tongue swelling
  - any difficulty breathing
  - wheeze
  - repetitive coughing
  - change in skin color (pale, blue)
  - sudden tiredness/lethargy/seeming limp

If you have any concerns about your infant’s response to peanut, seek immediate medical attention/call 911.

### Four recipe options, each containing approximately 2 g of peanut protein

*Note*: Teaspoons and tablespoons are US measures (5 and 15 mL for a level teaspoon or tablespoon, respectively).

*Option 1*: Bamba (Osem, Israel), 21 pieces (approximately 2 g of peanut protein)

*Note*: Bamba is named because it was the product used in the LEAP trial and therefore has proven efficacy and safety. Other peanut puff products with similar peanut protein content can be substituted.

- For infants less than 7 months of age, soften the Bamba with 4 to 6 teaspoons of water.
- For older infants who can manage dissolvable textures, unmodified Bamba can be fed. If dissolvable textures are not yet part of the infant's diet, softened Bamba should be provided.

*Option 2:* Thinned smooth peanut butter, 2 teaspoons (9–10 g of peanut butter; approximately 2 g of peanut protein)

- Measure 2 teaspoons of peanut butter and slowly add 2 to 3 teaspoons of hot water.
- Stir until peanut butter is dissolved, thinned, and well blended.
- Let cool.
- Increase water amount if necessary (or add previously tolerated infant cereal) to achieve consistency comfortable for the infant.

*Option 3*: Smooth peanut butter puree, 2 teaspoons (9–10 g of peanut butter; approximately 2 g of peanut protein)

- Measure 2 teaspoons of peanut butter.
- Add 2 to 3 tablespoons of previously tolerated pureed fruit or vegetables to peanut butter. You can increase or reduce volume of puree to achieve desired consistency.

*Option 4*: Peanut flour and peanut butter powder, 2 teaspoons (4 g of peanut flour or 4 g of peanut butter powder; approximately 2 g of peanut protein)

*Note*: Peanut flour and peanut butter powder are 2 distinct products that can be interchanged because they have, on average, a similar peanut protein content.

- Measure 2 teaspoons of peanut flour or peanut butter powder.
- Add approximately 2 tablespoons (6–7 teaspoons) of pureed tolerated fruit or vegetables to flour or powder. You can increase or reduce the volume of puree to achieve desired consistency.

## Appendix E. For health care providers: In-office supervised feeding protocol using 2 g of peanut protein

### General instructions

1. These recommendations are reserved for an infant defined in guideline 1 as one with severe eczema, egg allergy, or both and with negative or minimally reactive (1 to 2 mm) SPT responses and/or peanut sIgE levels of less than 0.35 kU_A_/L. They also may apply to the infant with a 3 to 7 mm SPT response if the specialist health care provider decides to conduct a supervised feeding in the office (as opposed to a graded OFC in a specialized facility [see Fig. 1]).
  These recommendations can also be followed for infants with mild-to-moderate eczema, as defined in guideline 2, when caregivers and health care providers may desire an in-office supervised feeding.
2. Proceed only if the infant shows no evidence of any concomitant illness, such as an upper respiratory tract infection.
  - Start with a small portion of the initial peanut serving, such as the tip of a teaspoon of peanut butter puree/softened Bamba.
  - Wait 10 minutes; if there is no sign of reaction after this small portion is given, continue gradually feeding the remaining serving of peanut-containing food (see options below) at the infant’s typical feeding pace.
  - Observe the infant for 30 minutes after 2 g of peanut protein ingestion for signs/symptoms of an allergic reaction.

### Four recipe options, each containing approximately 2 g of peanut protein

*Note*: Teaspoons and tablespoons are US measures (5 and 15 mL for a level teaspoon or tablespoon, respectively).

*Option 1*: Bamba (Osem, Israel), 21 pieces (approximately 2 g of peanut protein)

*Note*: Bamba is named because it was the product used in the LEAP trial and therefore has known peanut protein content and proven efficacy and safety. Other peanut puffs products with similar peanut protein content can be substituted for Bamba.

- For infants less than 7 months of age, soften the Bamba with 4 to 6 teaspoons of water.
- For older infants who can manage dissolvable textures, unmodified Bamba can be fed. If dissolvable textures are not yet part of the infant’s diet, softened Bamba should be provided.

*Option 2*: Thinned smooth peanut butter, 2 teaspoons (9–10 g of peanut butter; approximately 2 g of peanut protein)

- Measure 2 teaspoons of peanut butter and slowly add 2 to 3 teaspoons hot water.
- Stir until peanut butter is dissolved and thinned and well blended.
- Let cool.
- Increase water amount if necessary (or add previously tolerated infant cereal) to achieve consistency comfortable for the infant.

*Option 3*: Smooth peanut butter puree, 2 teaspoons (9–10 g of peanut butter; approximately 2 g of peanut protein)

- Measure 2 teaspoons of peanut butter.
- Add 2 to 3 tablespoons of previously tolerated pureed fruit or vegetables to peanut butter. You can increase or reduce volume of puree to achieve desired consistency.

*Option 4*: Peanut flour and peanut butter powder, 2 teaspoons (4 g of peanut flour or 4 g of peanut butter powder; approximately 2 g of peanut protein)

*Note*: Peanut flour and peanut butter powder are 2 distinct products that can be interchanged because they have, on average, a similar peanut protein content.

- Measure 2 teaspoons of peanut flour or peanut butter powder.
- Add approximately 2 tablespoons (6–7 teaspoons) of pureed tolerated fruit or vegetables to flour or powder. You can increase or reduce the volume of puree to achieve desired consistency.

## Appendix F. Peanut protein in peanut-containing foods

If the decision is made to introduce dietary peanut to the infant’s diet, the total amount of peanut protein to be regularly consumed per week should be approximately 6 to 7 g over 3 or more feedings. In the LEAP trial, at evaluations conducted at 12 and 24 months of age, 75% of children in the peanut consumption group reported eating at least this amount of peanut.

### Be aware of choking risks

- Whole nuts should not be given to children less than 5 years of age.
- Peanut butter directly from a spoon or in lumps/dollops should not be given to children less than 4 years of age.

If, after a week or more eating peanut, your infant or child displays mild allergic symptoms within 2 h of eating peanut, you should contact your health care provider.

Typical peanut-containing foods, their peanut protein content, and feeding tips for infants are provided in Table 2, and their nutritional content is found in Table 3.

**Table 2:** Typical peanut-containing foods, their peanut protein content, and feeding tips for infants

	Bamba	Peanut butter	Peanuts	Peanut flour or peanut butter powder
Amount containing approximately 2 g of peanut protein	17 g *or* % of a 28-g (1-oz) bag *or* 21 sticks	9–10 g *or* 2 teaspoons	8 g *or* ~10 whole peanuts (2½ teaspoons of grounded peanuts)	4 g *or* 2 teaspoons
Typical serving size	1 bag (28 g)	Spread on a slice of bread or toast (16 g)	2½ teaspoons of ground peanuts (8 g)	No typical serving size
Peanut protein per typical serving	3.2 g	3.4 g	2.1 g	No typical serving size
Feeding tips	For a smooth texture, mix with warm water (then let cool) or breast milk or infant formula and mash well Pureed or mashed fruit or vegetables can be added Older children can be offered sticks of Bamba	For a smooth texture, mix with warm water (then let cool) or breast milk or infant formula For older children, mix with pureed or mashed fruit or vegetables or any suitable family foods, such as yogurt or mashed potatoes	Use blender to create a powder or paste 2-2½ teaspoons of ground peanuts can be added to a portion of yogurt or pureed fruit or savory meal	Mix with yogurt or apple sauce

Bamba (Osem, Israel) is named because it was the product used in the LEAP trial and therefore has known peanut protein content and proven efficacy and safety. Other peanut puff products with similar peanut protein content can be substituted for Bamba

Teaspoons and tablespoons are US measures (5 and 15 mL for a level teaspoon or tablespoon, respectively)

**Table 3:** Nutritional content of peanut-containing foods

Per approximately 2 g of peanut protein	Bamba^a^ (17 g)	Peanut butter (10 g)	Peanuts (8 g)	Peanut butter powder (4 g)	Peanut flour (4 g)
kcal	93	59	45	15	13
Sugar (g)	0.4	0.65	0.38	0.4	0.33
Salt (mg)	68	48	1	31	7
Fat (g)	6.1	4.95	3.94	0.49	0.02

^a^The nutritional content of peanut puff products (other than Bamba) can be obtained from their manufacturers

## Appendix G. Graded OFC protocol

From “Conducting an oral food challenge to peanut in an infant: a work group report” [30].

### General instructions

1. A graded OFC should be performed only by a specialist with the training and experience to (1) perform and interpret skin prick testing and OFCs and (2) know and manage their risks. Such persons must have appropriate medications and equipment on site.
2. Four peanut preparations are provided:
  - *Option 1*: Smooth peanut butter mixed with either a previously tolerated pureed fruit or vegetable.
  - *Option 2*: Smooth peanut butter dissolved carefully with hot water and cooled.
  - *Option 3*: Peanut flour mixed with either a previously tolerated pureed fruit or vegetable. Peanut butter powder can be used instead of the peanut flour.
  - *Option 4*: Bamba peanut snack dissolved in hot water and cooled or even as a solid (ie, as a stick).
    *Note*: Bamba (Osem, Israel) is named because it was the product used in the LEAP trial and therefore has known peanut protein content and proven efficacy and safety. Other peanut puff products with similar peanut protein content can be substituted for Bamba.
3. The peanut protein content of the graded OFC protocol is identical for all peanut preparations provided below, except that the volume of food ingested per dose is different. The protocol consists of 5 incremental doses, given 15 to 20 min apart, with a cumulative peanut protein total of approximately 4 g per the 3.9 g total in the LEAP trial.
4. Refer to Table 4 and direct parents to discontinue specific medications for the prescribed amount of time before the graded OFC. Note that certain medications are allowed.
  **Table 4:** Medication discontinuation considerations before OFC
  Medications to be discontinued	Last dose before OFC
  Cetirizine	5 days
  Cyproheptadine	10 days
  Diphenhydramine	3 days
  Fexofenadine	3 days
  Loratadine	7 days
  Short-acting bronchodilator (eg, albuterol)	8 h
  Medications that can be continued	
  Antihistamine eye drops	
  Inhaled/intranasal corticosteroids	
  Topical (cutaneous) steroids	
  Topical (cutaneous) pimecrolimus, tacrolimus	

### Be prepared in case of a severe reaction (see Table 5)

*Note*: Teaspoons and tablespoons are US measures (5 and 15 mL for a level teaspoon or tablespoon, respectively).

**Table 5:** Emergency medications for a severe reaction during an office-based infant OFC

	Medication	Dose
First-line treatment	Epinephrine (1:1000 concentration)	0.01 mg/kg IM in the mid-outer thigh in health care settings *or* 0.15 mg of autoinjector IM in the mid-outer thigh in community settings Epinephrine doses may need to be repeated every 5-15 min
Adjunctive treatment	Albuterol nebulization	0.15 mg/kg every 20 min × 3 doses (minimum of 2.5 mg per dose) over 5-15 min
	Albuterol MDI inhalation	2 puffs, 90 μg per puff, with face mask
	Oxygen	8-10 L/min through a face mask
	Diphenhydramine	1.25 mg/kg administered orally
	Cetirizine	2.5 mg administered orally
	Normal saline (0.9% isotonic solution) or lactated ringers	20 ml/kg per dose administered over 5 min intravenously
	Steroids	Prednisolone 1 mg/kg administered orally *or* Solu-Medrol 1 mg/kg administered intravenously

*IM*, intramuscular; *MDI*, metered-dose inhaler

### Protocol instructions for options 1, 2, and 3 (see Tables 6, 7, and 8)

1. Measure peanut butter, peanut flour, or peanut butter powder for dose 1.
2. Prepare the first dose:
  - If using option 1, add previously tolerated pureed fruit or vegetable to measured dose 1 peanut butter and stir until well blended. You can increase or reduce volume of puree to achieve desired consistency. *Note*: Increasing the volume may increase the difficulty of getting through the entire protocol with a young baby.
  - If using option 2, slowly add hot water to measured dose 1 peanut butter and stir until peanut butter is dissolved, thinned, and well blended. Let the mixture cool. You can increase water volume (or add previously tolerated infant cereal) to achieve desired consistency.
  - If using option 3, add previously tolerated pureed fruit or vegetable to measured dose 1 peanut flour or peanut butter powder and stir until well blended. You can increase or reduce volume of puree to achieve desired consistency. *Note*: Increasing the volume may increase the difficulty of getting through entire protocol with a young baby.
3. Label dose 1.
4. Repeat steps 1 to 3 for the remaining doses 2 through 5, labeling each dose appropriately and before proceeding to the preparation of the next dose.
5. Feed dose 1 to infant and observe for symptoms of reactivity for 15 to 20 min.
6. If no symptoms appear, repeat with dose 2 and observe for 15 to 20 min.
7. Continue in this manner with doses 3, 4, and 5.

**Table 6:** Option 1: Measures for smooth peanut butter puree

Dose	Peanut butter volume^a^	Equivalent weight of peanut butter (g [peanut protein content in grams])^b^	Pureed fruit or vegetable volume	Total volume
1	1/8 teaspoon	0.67 (0.15)	1/2 teaspoon	5/8 teaspoon
2	1/4 teaspoon	1.33 (0.29)	3/4 teaspoon	1 teaspoons
3	1/2 teaspoon	2.67 (0.59)	1 teaspoons	1 1/2 teaspoons
4	1 teaspoon	5.33 (1.17)	2 teaspoons	3 teaspoons^c^
5	1 1/2 teaspoons	8 (1.6)	4 teaspoons	5 1/2 teaspoons
		Total protein: 3.96 g		

^a^Amounts (volume) of peanut butter measured as teaspoons are approximate measures to keep the dosing as practical as possible

^b^Peanut protein content is calculated on the average amount of protein for a range of butters using “Report: 16167, USDA Commodity, Peanut Butter, smooth,” from the USDA Nutrition Database (http://ndb.nal.usda.gov/ndb/foods)

^c^Three teaspoons = 1 tablespoon

**Table 7:** Option 2: Measures for smooth thinned peanut butter

Dose	Peanut butter volume^a^	Equivalent weight peanut butter (g [peanut protein content in grams])^b^	Volume of hot water	Total volume
1	1/8 teaspoon	0.67 (0.15)	1/8 teaspoon	1/4 teaspoon
2	1/4 teaspoon	1.33 (0.29)	1/4 teaspoon	1/2 teaspoon
3	1/2 teaspoon	2.67 (0.59)	1/2 teaspoon	1 teaspoon
4	1 teaspoon	5.33 (1.17)	1 teaspoon	2 teaspoons
5	1 1/2 teaspoons	8 (1.76)	1 1/2 teaspoons	3 teaspoons^c^
		Total protein: 3.96 g		

^a^Amounts (volume) of peanut butter measured as teaspoons are approximate measures to keep the dosing as practical as possible

^b^Peanut protein content is calculated on the average amount of protein for a range of butters using “Report: 16167, USDA Commodity, Peanut Butter, smooth,” from the USDA Nutrition Database (http://ndb.nal.usda.gov/ndb/foods)

^c^Three teaspoons = 1 tablespoon

**Table 8:** Option 3: Measures for peanut flour or peanut butter powder

Dose	Peanut flour or peanut butter powder volume^a^	Equivalent weight peanut flour or peanut butter powder^b^ (g [peanut protein content in grams])	Pureed fruit or vegetable volume	Total volume
1	1/8 teaspoon	0.25 (0.13)	1/2 teaspoon	3/4 teaspoon
2	1/4 teaspoon	0.5 (0.25)	1 teaspoon	1 1/4 teaspoons
3	1/2 teaspoon	1.0 (0.5)	2 teaspoons	2 1/2 teaspoons
4	1 teaspoon	2.0 (1.0)	3 teaspoons^c^	4 teaspoons
5	2 teaspoons	4.0 (2.0)	6 teaspoons^d^	8 teaspoons
		Total protein: 3.88 g		

^a^Amounts (volume) of peanut flour or peanut butter powder measured as teaspoons are approximate measures to keep the dosing as practical as possible

^b^Information regarding peanut powder and flour reflects averages obtained from the producers. Most brands of peanut flour/peanut butter powder are approximately 50% peanut protein by weight. However, weight can vary based on the fat content and also the brand chosen. Therefore a weight measurement can be more accurate than household measurements

^c^Three teaspoons = 1 tablespoon

^d^Six teaspoons = 2 tablespoons

### Protocol instructions for option 4 (see Table 9)

1. Count Bamba sticks for dose 1.
2. Prepare the first dose by slowly adding hot water to measured Bamba and stirring until Bamba is dissolved, thinned, well blended, and cooled. You can increase water volume to achieve desired consistency. *Note*: Increasing the volume may increase the difficulty of getting through the entire protocol with a young baby.
3. Label dose 1.
4. Repeat steps 1 to 3 for the remaining doses 2 through 5, labeling each dose appropriately and before proceeding to the preparation of the next dose.
5. Feed dose 1 to the infant and observe for symptoms of reactivity for 15 to 20 min.
6. If no symptoms appear, repeat with dose 2 and observe for 15 to 20 min.
7. Continue in this manner with doses 3, 4, and 5.

**Table 9:** Option 4: Bamba peanut snack (Osem, Israel)

Dose	Bamba, no. of sticks	Equivalent weight (peanut protein content [g])^a^	Volume of hot water (approximate, will need to be adjusted for each child)	Approximate final volume
1	1 stick	0.81 (0.1)	½ teaspoon	¾ teaspoons
2	3 sticks	2.43 (0.3)	1 teaspoon	1½ teaspoons
3	5 sticks	4.05 (0.5)	1½ teaspoons	2¼ teaspoons
4	10 sticks	8.1 (1.0)	3 teaspoons	4 teaspoons
5	21 sticks	17.01 (2.0)	6 teaspoons	7½ teaspoons
		Total protein: 3.9 g		

Other peanut puffs products with equivalent peanut protein content can be substituted for Bamba

^a^The amount of Bamba sticks is an approximate measure looking at a range of Bamba products. Bamba snacks from different parts of the world have a varied peanut protein content [30]. The peanut protein content of Bamba was calculated according to the publication by Du Toit et al. [13]

## Abbreviations

CC: Coordinating Committee
EP: expert panel
GRADE: Grading of Recommendations Assessment, Development and Evaluation
LEAP: Learning Early about Peanut Allergy
NIAID: National Institute of Allergy and Infectious Diseases
OFC: oral food challenge
sIgE: specific IgE
SPT: skin prick test
